# Medicalisation of vaping in the UK? E-cigarette users’ perspectives on the merging of commercial and medical routes to vaping

**DOI:** 10.1177/17579139231185481

**Published:** 2023-08-06

**Authors:** E Ward, L Dawkins, R Holland, I Pope, C Notley

**Affiliations:** University of East Anglia, Norwich Research Park, Norwich NR4 7TJ, UK; London South Bank University, London, UK; University of Leicester, Leicester, UK; University of East Anglia, Norwich, UK; University of East Anglia, Norwich, UK

**Keywords:** e-cigarettes, intervention, qualitative, smoking, vaping industry, healthcare professionals

## Abstract

**Background:**

In the UK, most smokers choosing e-cigarettes to quit smoking will access vaping via commercial routes. In recent years, however, a shift towards medicalisation of vaping has become apparent, with public health guidance supporting e-cigarettes for smoking cessation and increased partnership working between healthcare professionals and the vaping industry. To achieve the UK’s Smokefree 2030 target, the UK Government has set out measures to use e-cigarettes in National Health Service (NHS) settings and to move towards streamlining processes to make e-cigarettes available to a million smokers. This article aims to understand acceptability of different approaches by seeking perspectives of people with lived experience of e-cigarette use for smoking cessation.

**Methods:**

Mixed methods data collected between March 2018 and March 2019 as part of a broader study of e-cigarette use trajectories (ECtra study). Data here relate to the views of partnership working and medicalisation of vaping extracted from 136 interviews/extended surveys of people who had used e-cigarettes to try to stop smoking. Qualitative data were thematically analysed. Participant ratings of interventions were presented descriptively, and differences in participant characteristics and ratings were reported.

**Results:**

Three qualitative themes were identified: pro-partnership, anti-partnership and medicalisation dissonance. Medicalisation was discussed for its potential to reassure smokers about e-cigarette harms and its potential to reach smokers from disadvantaged backgrounds. Concerns were raised about cost-effectiveness, quality of support, conflicts of interest and limiting product choice. Most participants rated interventions involving partnership working as potentially helpful in switching from smoking to vaping. There were no statistically significant associations between age, gender and socioeconomic status, and helpfulness ratings.

**Conclusion:**

Both commercial and medical routes to vaping offer perceived benefits to vapers and may complement and reinforce each other to support smoking cessation.

## Introduction

Since e-cigarettes emerged as a ‘disruptive’ consumer technology,^1^ two routes of access for people wishing to quit smoking via vaping have materialised. Access via purchasing from commercial markets is by far the most popular. Less common is access via healthcare professionals (HCPs), and only Australia has limited access to vaping products via this route, with recent legislation requiring all purchases of nicotine vaping products to require a prescription.^2^ In the UK, both medical and commercial approaches have been embraced due to public health bodies acknowledging vaping’s potential for assisting smoking cessation and supporting e-cigarette use for this purpose.^3^ A Cochrane living systematic review^4^ of randomised controlled trials suggests clear evidence that e-cigarettes are twice as effective compared to other forms of smoking cessation support, such as nicotine replacement therapy (NRT).

Most smokers try vaping without seeking or receiving support from HCPs. The UK commercial vaping sector is the most popular place for the public to purchase e-cigarettes, with 94% of vapers in 2021 accessing products via vape shops, online, garages, supermarkets and convenience stores.^5^ While there is widespread accessibility to these products, it is important to recognise that the industry has been regulated in the UK via European Union legislation for safety since 2014.^6^ Currently, e-cigarettes thrive as a commercial product in the UK, but vaping has also been medicalised to an extent, as evidenced by the implementation of National institute for health and care excellence (NICE) guidelines^7^ recommending HCPs support patients’ choice to use e-cigarettes for cessation; medicines and healthcare products regulatory agency (MHRA) guidance^8^ to support licencing e-cigarettes as medicines; increasing numbers of Stop Smoking Services (SSS) becoming ‘e-cigarette friendly’^9^ with 40% of SSS facilitating free access to e-cigarettes^10^; targeted interventions based in primary and secondary care including provision of e-cigarettes^4^ and e-cigarettes being available for sale in hospitals and pharmacies.

Despite the public health support of e-cigarettes as a cessation tool, many HCPs remain cautious about supporting patients who smoke to make a quit attempt using e-cigarettes due to a lack of knowledge and confidence in discussing the effectiveness and relative harms of vaping,^11–14^ likely resulting in potential quitters not receiving the help they need.^15^ Conversely, other HCPs have actively supported patients to use e-cigarettes, with some GPs and SSS forming partnerships with the vaping industry, capitalising on their ‘expertise by experience’.^16,17^ These partnerships include informal referrals and signposting to local vape shops by HCPs,^16^ vape shop staff receiving smoking cessation training to deliver smoking cessation support in-house^18–20^ and HCPs providing patients with a ‘vaping starter kit’^21,22^ or giving vouchers to redeem in local vape shops.^23^ There is limited evaluation of the effectiveness of such partnerships (especially regarding informal referrals and vape shop training). Examples include 1022 residents in a deprived inner-city area given a starter kit via pharmacies and SSS resulting in a 4-week quit rate of 37.4%,^21^ and 668 smokers predominantly resident in a deprived English seaside town, who had previously failed to quit smoking using traditional methods, provided with a vape shop voucher by the local SSS resulting in a 4-week quit rate of 21%.^23^ These studies of real-world practice reinforce randomised controlled trial results^4^ which indicate that interventions that provide access to e-cigarettes may be effective in helping people to quit and suggest that e-cigarettes might be particularly effective for disadvantaged groups.

The UK Government hopes to achieve a national target of England being smokefree by 2030 (defined as 5% smoking prevalence or less). The recent independent Khan review^24^ recommended that to achieve the Smokefree 2030 target, the Government should accelerate the path to e-cigarettes being available on prescription and provide free vape starter kits to people from deprived communities. The report also recommended that brief smoking cessation advice should be delivered routinely in all National Health Service (NHS) settings and that HCPs should be fully informed about the benefits for patients of switching to vaping. Following these recommendations, in April 2023, the Department of Health and Social Care (DHSC) announced a variety of measures, including offering stop smoking support to all smokers admitted to hospital and a ‘swap to stop’ vaping starter kit scheme targeting 1 million smokers with the aim of reducing health inequalities.^25^ The proposed measures mean that opportunities to access e-cigarettes via medicalised routes, such as via SSS, will increase in the lead up to 2030. Equally, the UK e-cigarette market share is expected to increase by US$1.20 billion from 2021 to 2027.^26^ Given the likely increase in both the medical and commercial routes to vaping, it is important that we seek the views of people with lived experience of attempting to quit smoking using e-cigarettes to understand the acceptability and potential cessation efficacy of the different approaches. This article aims to answer the research question: ‘How helpful do UK vapers perceive partnership working between HCPs and the vaping industry in supporting people to stay stopped from smoking?’

## Methodology

The data drawn upon to answer the research question are taken from the second phase of a broader longitudinal study, the ‘E-Cigarettes Trajectories Study’ (ECTra), exploring patterns of e-cigarette use in preventing smoking relapse through longitudinal mixed methods data collection. The study received ethical approval from the UEA Faculty of Medicine and Health Sciences Research Ethics Committee (project reference: 2017/2018 – 106).

### Sample

Between March 2018 and March 2019, 184 participants took part in the second phase of the ECTra study, 12–18 months after they initially participated in the first phase (2016–2017). The eligibility criteria included adults (18 years and over) who had attempted to use an e-cigarette for smoking cessation. Participants were originally recruited into the first phase of the study through word of mouth, social media, local press articles, vape shops and university bulletins. The ECtra study was initially designed to be an interview study, but due to over recruitment, the research team offered an alternative online survey version of the interview. This was administered via a hyperlink using the Qualtrics survey platform to enquirers who were unable to participate in an interview. Participants gave informed consent before taking part in the confidential online survey (147) or telephone (25)/face-to-face (12) interview. To address the current research question, which focuses on the perspectives of practice potentially facilitating UK policy changes, people resident outside of the UK were excluded from the analysis. In total, 11 UK participants who did not provide any data relating to the research question were also excluded, resulting in a final sample of 136 (37 interviewed and 99 surveyed).

### Procedure

Phase 2 online survey and interview topic guide were developed in consultation with lay consultants (Supplementary Material). The questions were derived from the findings illuminated from the first phase of the ECtra study and explored practices identified since the first phase. Both data collection tools included a questionnaire listing 14 examples of interventions involving partnership working between the vaping industry and HCPs. These examples were developed by the research team and included examples of practice already in existence, practice proposed by public health bodies or HCPs, and ideas influenced by participants’ responses to the first phase of the study (Supplemental Material). Participants were asked to rate how helpful each would have been for them, or someone else, to stay stopped from smoking using an e-cigarette on a five-point scale (*not at all helpful* to *extremely helpful*). This article focuses on reporting the results for the three common partnership practices in the UK: (1) healthcare practitioner signposting to a vape shop, (2) vape shop voucher schemes, (3) in-house vape shop smoking cessation behavioural support and (4) the possible plans for e-cigarettes to be available on prescription. The questionnaire was followed by open-ended questions (text box in the questionnaire) inviting participants to explain their answers and offer their opinions. Both data collection tools included the same question phrasing (Supplementary Material).

### Analysis

Interviews were recorded, transcribed verbatim and anonymised. Survey data were downloaded once the survey closed. Participant’s responses to the open-ended questions were extracted from interview transcripts and downloaded survey data, then were uploaded to NVivo 12 qualitative analysis software. The qualitative extracts from both data collection tools were combined and coded by E.W. using a standardised inductive thematic analysis method.^27^ C.N. then coded 10% of extracts to ensure intercoder reliability.

Quantitative questionnaire data from both data collection tools were entered into SPSS. These data were analysed descriptively. Exploratory analysis was undertaken by dichotomising helpfulness ratings to investigate the characteristics of participants who stated that they would have most benefitted from the different interventions: this was measured by collapsing *very helpful* and *extremely helpful* ratings into one category, and *not at all, slightly* and *somewhat* helpfulness scores into another. Pearson’s chi-square analyses were used to investigate associations between helpfulness ratings of the four practice examples and gender, and socioeconomic status (SES), measured by collapsing participants into two groups: managerial/professional/technical (A-C1) versus routine and manual/students/retired/unemployed (other groups). Independent *t*-tests were used to investigate differences in mean age between the helpfulness rating dyads. The variables were analysed together using logistic regression for each of the interventions to predict characteristics of those participants rating the intervention as *very/extremely helpful*.

## Results

The profile of participant characteristics is reported in Table 1. Just over a quarter were female (38, 27.9%), ages ranged from 22 to 79 years (M 49.5, SD 12.6), three participants were from ethnic minority backgrounds and 50% (68) were employed in managerial, professional or technical occupations. Most participants were vaping and abstinent from tobacco (117, 86%), 10 participants had relapsed (4 dual using both tobacco and vaping) and 9 were no longer using either e-cigarettes or tobacco.

**Table 1:** Profile of participant characteristics (***n*** = 136)

	Sample^a^
Gender:	
Male	72.1% (98)
Female	27.9% (38)
Age:	
Range (years)	57: 22–79
Mean (years)	49.5 (SD 12.6)
Ethnicity (*n* = 133)	
White	97.7% (130)
Ethnic minorities	1.5% (3)
Managerial, professional or technical occupation:	50% (68)
T2 vaping status	
Vaping and abstinent from tobacco	86% (117)
Abstinent from both vaping and tobacco	6.6% (9)
Relapsed to tobacco (dual using)	2.9% (4)
Relapsed to tobacco (not vaping)	4.4% (6)

SD: standard deviation.

a Participants identifying as resident in the UK who answered the medicalisation questions.

## Qualitative Findings

Table 2 shows a summary of the inductive thematic analysis. Subthemes were identified in the data, centring around ethics, suitability, accessibility, impact on NHS and vaping industry. These were grouped into three overarching themes: ‘pro-partnership’, ‘anti-partnership’ and ‘medicalisation dissonance’. They are discussed in turn below.

**Table 2:** Thematic analysis of participants’ responses to open-ended questions about partnership working and medicalisation

Subthemes	Pro-partnership	Anti-partnership	Medicalisation dissonance
Ethics	Provide reassurances about health impacts, device safety and quality of advice	Concerns about e-cig long-term safety and normalisation of nicotine	Lead to further regulation which could stifle product development, choice and independent sector
Suitability	Could help disadvantaged groups	Personal choice to vape and health own responsibility	Vaping is pleasurable and not just for smoking cessation
Accessibility	Simplify vaping, reduce intimidation and increase affordability	Open to fraud and abuse	Already effective and affordable
NHS impacts	Cost-effective for NHS as preventative	Increase financial burden on NHS	NHS promotion of vaping, rather than intervention
Vaping industry impacts	Need for quality assurance	Commercial interests	Unfair responsibility

### Pro-partnership

Some participants expressed themes that supported partnership working, such as believing it would offer smokers reassurances about vaping health risks, safety issues and the quality of advice given in vape shops:

*Depending on the smoker, information given by a health professional helps to give them that last push to convince them that this is an option for them. I believe training vape shop employees in the basics of smoking cessation to give clients tips and support for identifying triggers and helping them remain abstinent not only strengthens the relationship between the (soon to be ex) smoker and their choices, but also helps boost confidence levels. (Survey participant 115)*

They felt that the medicalisation of vaping could simplify the idea of vaping and reduce intimidation and could make initiating vaping more affordable for lower socioeconomic groups. Comparisons were made to NRT being available on prescription, and that partnership working had the potential to prevent more disease and morbidity and was therefore cost-effective:

*How would it affect the budget of the NHS? If it’s helping people become more healthy, there are fewer people who are going to need heart operations and help with lung problems. It would help people in less advantageous financial conditions. (Interview participant 20)*

A few participants did raise concerns that there would need to be mechanisms for ensuring consistency across services and interventions to ensure quality of support.

### Anti-partnership

Some participants expressed themes that were against partnership working, such as concerns that approaches could be open to fraud and abuse with people potentially taking advantage of prescriptions or voucher schemes. A common view was that NHS budgets were under pressure and that e-cigarette interventions would add to the financial burden:

*I suppose that could be quite a draining resource on the NHS if people just think ‘oh well that’s free, I’ll have it, I’ll try it’ and then don’t actually commit to it. (Interview participant 39)*

A few felt that people should be responsible for their own health and that vaping was a personal choice rather than a medicalised treatment option. There were concerns around potential conflicts of interest in involving an industry with commercial interests. Ethical arguments were put forward including potential for vaping to normalise nicotine use in young people and not knowing the long-term health impacts of e-cigarettes:

*If, in 20 years’ time, all results around vaping suggests that, like it doesn’t cause cancer, but it causes aneurysms, then you know the industry has been built up and like supported by the NHS. That’s going to feel pretty uncomfortable, particularly when their lobbyists try to prevent legislation being passed against it, in the same way that’s happened with tobacco. (Interview participant 27)*

### Medicalisation dissonance

Some participants expressed themes centring around a belief that vaping should be predominantly commercial and should not be medicalised because vaping had proved itself to be affordable and effective, and that support to help people quit was already being offered informally in vape shops:

*[Millions of] people are now using e-cigarettes in the UK and that this change came about with no involvement of any health professionals whatever. My guess is that the best thing would be for health professionals to leave things as they are, while doing everything they can to counteract adverse media reportage and trumpet the benefits of switching from smoking to e-cigarette use. (Survey participant 21)*

They commented that smoking should not be viewed as an illness and that e-cigarettes were ‘more than’ smoking cessation devices. Fears were expressed that partnership working could lead to further regulation which would stifle product development and reduce the pleasurable aspects of vaping (such as flavours, modifying and collecting devices), alongside negatively impacting on small independent vaping businesses and allowing vaping to be monopolised by the tobacco and pharmaceutical industries:

*It works because it’s a consumer product. For a start [ecig prescription] would drastically reduce the amount of products available, advances in equipment would stagnate and I believe it would become less effective. (Survey participant 26)*

Some commented that it was unfair to expect the vaping industry to support delivery of health interventions and that dialogue and knowledge transfer would be a better approach to take.

## Questionnaire Findings

**Figure 1:**
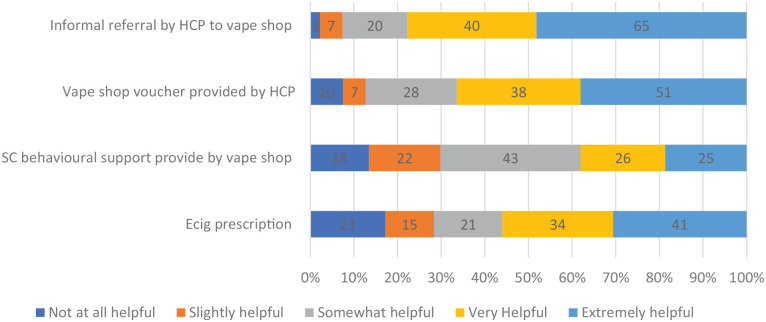
Proportion and number of participants rating each partnership approach on a five-point scale of helpfulness for stopping smoking (***n*** = 135)

Figure 1 reports the proportion and number of participants who endorsed each of the five categories of helpfulness for each of the four practice examples. Informal referral from a healthcare practitioner to a local vape shop was the most popular, with over three-quarters (77.7%) stating it to be very or extremely helpful in supporting themselves or others to stop smoking. Two-thirds (66.5%) stated that receiving a voucher from an HCP to spend at a local vape shop would have been very or extremely helpful. Over half (56%) stated that the availability of e-cigarettes on prescription would have been very or extremely helpful, but e-cigarettes on prescription also had the largest proportion of participants who stated that it would not have been at all helpful (17.2%). Smoking cessation support being provided in-house by vape shops had a more mixed response with 38.1% stating very or extremely helpful.

Gender, age and SES of the participants who stated that they would have most benefitted from the different interventions were investigated. Chi-square tests (Table 3) and *t-*tests (Table 4) demonstrated that helpfulness ratings between those who rated interventions as *very/extremely helpful* compared to those who rated them as *not at all/slightly/somewhat helpful* did not vary by age, gender and SES for any of the four interventions. In line with the finding of no associations on bivariate analyses, when all variables were analysed together using logistic regression (Table 5), again no single variable was found to have a relationship with the outcome explored (e.g. whether informal referral to a vape shop by a healthcare practitioner was considered very/extremely helpful or not) when adjusted for differences in the other two variables.

**Table 3:** Comparison of participants rating the interventions as no to somewhat helpful with those who rated interventions very/extremely helpful

	No/slightly/somewhat helpful rating		Very/extremely helpful rating		Chi-square	*p*
	*n*	%	*n*	%		
Informal referral by HCP to vape shop						
Gender						
Male	17	17.5	80	82.5	.666	.414
Female	9	23.7	29	76.3		
SES						
A-C1	11	16.2	57	83.8	1.099	.294
Other groups	15	23.4	49	76.6		
Vape shop voucher provided by HCP						
Gender						
Male	32	33.3	64	66.7	.009	.923
Female	13	34.2	25	65.8		
SES						
A-C1	23	33.8	45	66.2	.004	.953
Other groups	21	33.3	42	66.7		
E-cig available on prescription						
Gender						
Male	45	46.4	52	53.6	.795	.373
Female	14	37.8	23	62.2		
SES						
A-C1	31	45.6	37	54.4	.099	.753
Other groups	27	42.9	36	57.1		
Smoking cessation behavioural support provided by vape shop						
Gender						
Male	63	64.9	34	35.1	1.348	.246
Female	20	54.1	17	45.9		
SES						
A-C1	46	67.6	22	32.4	1.541	.241
Other groups	36	57.1	27	42.9		

HCP: healthcare professional; SES: socioeconomic status.

**Table 4:** Comparison of mean age of participants rating the interventions as not at all/slightly/somewhat helpful with those who rated interventions very/extremely helpful

	*n*	*df*	Not to somewhat helpful		Very/extremely helpful		*t*	*p*	Cohen’s *d*
			M	SD	M	SD			
Age (years) of participants rating:									
Informal referral by HCP to vape shop	135	133	47.9	13.842	49.8	12.369	−.675	.501	−.147
Vape shop voucher provided by HCP	134	132	49.4	12.078	49.2	12.905	.081	.936	.051
E-cig available on prescription	134	132	51.6	11.927	47.6	12.959	1.830	.069	.318
Smoking cessation behavioural support provided by vape shop	134	132	50.04	12.391	48.2	13.082	.808	.420	.144

SD: standard deviation; HCP: healthcare professional.

**Table 5:** Predicting participants rating the interventions as very/extremely helpful

	*N*	*df*	Chi-square	B (SE)	Adj odds ratio (95% CI)	*p*	*R* ^2^
Informal referral by HCP to vape shop	132	3	2.435				.12^a^ .02^b^ .03^c^
Constant				.99 (.88)	.2.701	.26	
Gender (female)				−.27 (.48)	.590 (.297–1.948)	.57	
SES (A-C1)				−.53 (.46)	.590 (.240–1.447)	.25	
Age (years)				.02 (.02)	1.016 (.982–1.051)	.36	
Vape shop voucher provided by HCP	131	3	.159				.90^a^ .00^b^ .00^c^
Constant				.92 (.78)	2.513	.24	
Gender (female)				−.12 (.42)	.888 (.394–2.005)	.78	
SES (A-C1)				.06 (.39)	1.066 (.499–2.280)	.87	
Age (years)				−.01 (.02)	.995 (.966–1.026)	.76	
E-cig available on prescription	131	3	4.377				.60^a^ .03^b^ .04^c^
Constant				1.31 (.78)	3.696	.09	
Gender (female)				.36 (.41)	1.428 (.636–3.209)	.39	
SES (A-C1)				.26 (.38)	1.301 (.619–2.731)	.49	
Age (years)				−.03 (.02)	.974 (.945–1.004)	.09	
Smoking cessation behavioural support provided by vape shop	131	3	3.633				.97^a^ .027^b^ .037^c^
Constant				−.04 (.76)	.962	.96	
Gender (female)				.30 (.41)	1.345 (.603–3.001)	.47	
SES (A-C1)				.53 (.38)	1.706 (.808–3.602)	.16	
Age (years)				−.02 (.02)	.983 (.955–1.013)	.26	

SE: standard error; CI: confidence interval; HCP: healthcare professional; SES: socioeconomic status.

a Hosmer and Lemeshow.

b Cox and Snell.

c Nagelkerke.

## Discussion

The majority of e-cigarette users stated that interventions including informal referrals by HCP to vape shops, and vape shop voucher schemes, would have been very/extremely helpful for them or someone else to have stay stopped from smoking using an e-cigarette. This is perhaps unsurprising given it is likely that the participants would have accessed vaping via a commercial route with most having achieved tobacco abstinence. Even so, half of those surveyed stated that more medicalised interventions, such as e-cigarettes being available on prescription, and smoking cessation behavioural support offered in vape shops, would have been very/extremely helpful. These findings were not related to gender, age or SES, perhaps indicating a broad acceptability of such interventions. A range of views given on the acceptability of commercial and medical routes were discussed. Common concerns were around cost-effectiveness, yet we know from trials that e-cigarettes can be a cost-effective treatment.^4^ There were also concerns about the conflicts of medical and commercial interests and the quality of support via partnership working that could be offered as a result. Existing schemes, however, have been shown to be feasible and potentially effective^21–23^ and vape shops have been acknowledged to play an important supportive role in helping people to stop smoking.^16,17^

Choice in vaping products is known to be important in switching success^28,29^ and there were concerns raised by participants that the interventions, especially provision of starter kit or e-cigs via prescription, would stifle the sector and limit choice. Trials which are similar to the prescription model, where HCPs give participants one specific e-cigarette, have been shown to be effective.^4^ It cannot be ignored, however, that thousands of people are estimated to quit using e-cigarettes bought in the commercial sector each year^30^ where they have an abundance of choice. Quitters often attribute their success with vaping due to being able to experiment with different products to find the vaping set-up that offers the most satisfaction.^28,31^ For some smokers who may not want the pressure of a formal quit attempt, vaping is appealing because it is a commercial product separate from medical intervention. Indeed, some vapers are ‘accidental’ quitters who did not set out to stop smoking but grew to prefer vaping.^32^ In addition, it is highly likely that those who start vaping by being given a specific e-cigarette by an HCP are going to have to engage with the vaping industry to buy consumables to continue vaping and avoid relapse. However, nearly a third of smokers still believe that vaping is more or equally harmful to health^33^ and the potential health risks were raised as a concern even in this group of predominantly successful switchers. As suggested by the participants, provision of e-cigarettes by HCPs, alongside other forms of partnership working, could offer smokers reassurances about the safety of e-cigarettes in comparison to tobacco. For the reasons outlined above, to maximise quitters’ chances of success, policymakers should carefully consider the impact of future regulations on limiting or restricting choice to ensure there is a wide range of different approaches to meet different needs. The education for HCPs proposed in the Khan Report may benefit from including not only the health benefits of switching but outlining factors that could help quitters succeed, such as seeking support from a reputable vape shop selling regulation compliant products.

Another concern raised by participants was that e-cigarettes available on prescription could inadvertently allow the tobacco industry to monopolise the sector. This concern has also been raised by policymakers and academics, who believe that only the tobacco companies will have the resources available to successfully undertake the licencing process.^34–36^ The UK government is party to the WHO Framework Convention on Tobacco Control (FCTC)^37^ and is committed to preventing the tobacco industry benefitting from tobacco control policies.^38^ E-cigarettes provided in trials and existing SSS practice are usually selected in part because they are produced by the independent vaping industry. Identifying independent products can be challenging, and to date, researchers^39^ and SSS have had to undertake their own due diligence (such as consulting with the Independent British Vape Trade Association^40^ and established independent companies). In response to the new DHSC measures including the ‘swap to stop’ scheme, a central procurement point has been set up allowing local authorities and SSS to buy e-cigarettes supplied by companies who have gone through extensive processes to check compliance with existing regulations,^6^ that they are good value for money and will state any conflicts of interest including tobacco company involvement.^41^ Using this route to purchase starter kits will not be mandatory but will save individual local authorities doing their own compliance checks and should ensure that FCTC is not breached.

### Limitations

Although the sample can be considered large for a qualitative study, the sample size is relatively modest for a survey, meaning quantitative analysis may be underpowered to detect effects and can therefore only be considered exploratory. The findings derived from a convenience sample may not be generalisable to the wider UK e-cigarette user population, and there was overrepresentation of white men, although it is interesting that statistical results did not vary by age, gender or SES. These exploratory results are helpful in guiding further research to ascertain who would benefit most from the different approaches.

The sample was not originally recruited to answer this specific research question on partnership working. However, the research question around interventions was included in Phase 2 following Phase 1 analysis highlighting the importance of SSS and the vaping industry and developments in policy and practice in this area since Phase 1. The qualitative data generated via verbal interview were generally richer than data generated via the survey, although the same themes were identified through triangulation. It was also beyond the scope of the study to obtain the views of smokers, although it would be very helpful to explore the potential impact of medicalisation on this group given they would be the targets of future interventions. Likewise, it would be helpful to explore the views of HCPs.

It should be noted that data were collected before widely publicised events which could have influenced responses including the ‘E-cigarette, or Vaping Product, Use-Associated Lung Injury’ (EVALI) outbreak in the US,^42^ the COVID 19 pandemic and publication of recent evidence.^43^ In addition, data were collected before the emergence of disposable vapes as a dominant presence in the UK e-cigarette market^44^ leading to the recent concern about disposable e-cigarette use among young people,^45^ and, therefore, themes presented may not reflect current trends and discourses. However, discourses surrounding vaping positioning ‘harm reduction for smokers’ arguments versus concerns about ‘prevention of harm from addiction for children/adolescents’ were prevalent before disposables emerged,^46^ and were reflected in the themes presented in this article.

## Conclusion

This research suggests that, from e-cigarette users’ perspectives, medical routes to vaping potentially offer some benefits in terms of reassurance about safety and additional support, but the implementation of interventions should not limit consumer choice as different approaches satisfy different needs for adult smoking cessation and personal preferences. There are concerns, however, about the increase in youth vaping, and measures have been suggested to restrict marketing practices to make the products less appealing to children.^47^ Any future tobacco control measures involving e-cigarettes need to be evaluated for both their impact on smoking cessation and prevention of youth uptake of vaping. Medical routes to vaping were acceptable to this group of current, predominantly exclusive vapers and were perceived to be potentially of help if they had been available when they were attempting to quit smoking. This supports the implementation of the proposed ‘swap to stop’ scheme, which will be the biggest smoking cessation scheme involving the English healthcare sector and the vaping industry to date. Further evaluation is needed to establish how local authorities can best implement the scheme (in terms of product choice and delivery methods) to target smokers from minority and disadvantaged groups where there is the highest smoking prevalence.

## Supplemental Material

sj-docx-1-rsh-10.1177_17579139231185481 – Supplemental material for Medicalisation of vaping in the UK? E-cigarette users’ perspectives on the merging of commercial and medical routes to vaping Supplemental material, sj-docx-1-rsh-10.1177_17579139231185481 for Medicalisation of vaping in the UK? E-cigarette users’ perspectives on the merging of commercial and medical routes to vaping by E Ward, L Dawkins, R Holland, I Pope and C Notley in Perspectives in Public Health

sj-docx-2-rsh-10.1177_17579139231185481 – Supplemental material for Medicalisation of vaping in the UK? E-cigarette users’ perspectives on the merging of commercial and medical routes to vaping Supplemental material, sj-docx-2-rsh-10.1177_17579139231185481 for Medicalisation of vaping in the UK? E-cigarette users’ perspectives on the merging of commercial and medical routes to vaping by E Ward, L Dawkins, R Holland, I Pope and C Notley in Perspectives in Public Health
