# Supplementary material for: Medicalisation of vaping in the UK? E-cigarette users’ perspectives on the merging of commercial and medical routes to vaping
Source: Perspect Public Health. 2023 Aug 6;145(5):277–87. doi: 10.1177/17579139231185481 (PMC12457728; doi:10.1177/17579139231185481)
Supplement: sj-docx-1-rsh-10.1177_17579139231185481 – Supplemental material for Medicalisation of vaping in the UK? E-cigarette users’ perspectives on the merging of commercial and medical routes to vaping [file sj-docx-1-rsh-10.1177_17579139231185481.docx]

**Supplementary material 1**

We have some potential ideas of how the vaping community and health professionals could work together.

For each idea listed below, I would like you to rate on a scale of 1-5 how helpful the idea has been or would have been for you, or someone else, trying to stay stopped from smoking using an e-cigarette.

Please write the corresponding number in the box next to each statement:

1 = not at all helpful

2 = slightly helpful

3 = somewhat helpful

4 = very helpful

5 = extremely helpful

| A peer-support scheme where a new user can seek advice from a more experienced vaper |  |
| --- | --- |
| Receiving written information (e.g. leaflet, website) about the advantages and disadvantages about the different types of devices and information of how to use e-cigarettes from your doctor or stop smoking service |  |
| Doctor, Stop Smoking Service advisor or other health professional referring a person looking to give up smoking using an e-cigarette to a reputable vape shop for advice on vaping products and using them effectively |  |
| A voucher scheme – for example, being given a voucher by doctor to spend on e-cigarette products in a reputable vape shop |  |
| Discounts for clear CO testing – for example, receiving a discount in shops by proving you have remained abstinent from smoking for a period |  |
| Starter kits available on prescription |  |
| Receiving free ongoing support from shops with vaping such as trouble shooting issues with devices, device maintenance, and changing consumables |  |
| Vape shop staff outreach – for example, vape shop staff holding a session at a Stop Smoking Service to explain to staff and clients different vaping products and demonstrate how to use them |  |
| Vape shop staff outreach to the public – for example, a mobile vape shop and Stop Smoking Service to visit deprived or remote areas of the country |  |
| Being offered written health information (e.g. leaflet) about stopping smoking and vaping when purchasing an e-cigarette |  |
| Receiving health information about up giving up smoking from a vape shop |  |
| Receiving smoking cessation behavioural support from a vape shop such as goal setting or identifying lapse triggers |  |
| Receiving information from shops about research into safety and health risks of vaping |  |
| A ‘kite mark’ displayed in shops to show they have undergone smoking cessation training ensuring information given was evidence based |  |

What do you think about health professionals working with the vaping community, such as the ideas proposed above?  What are the advantages? What are the disadvantages?  What sort of information would you have liked from health professionals about vaping?  What sort of information would you have liked from vape retailers about quitting smoking and staying stopped?   

What do you think about further medicalisation of vaping (e.g. medicinal licences for vaping products, licences for retailers, production regulations, etc.)? What are the advantages? What are the disadvantages? How can the commercial interests of industry be balanced against the health promotion interests of health bodies?
